# Opinions of the Urban Women on Pap Test: Evidence from Bangladesh

**DOI:** 10.31557/APJCP.2019.20.6.1613

**Published:** 2019

**Authors:** Sohela Mustari, Belal Hossain, Nurazzura Mohamad Diah, Susmita Kar

**Affiliations:** 1 *Department of Sociology, Begum Rokeya University, Rangpur, *; 2 *Directorate General of Health Services (DGHS) Mohakhali, *; 4 *Department of Public Health, Faculty of Allied Health Science, Daffodil International University, Dhaka, Bangladesh, *; 3 *Department of Sociology and Anthropology Kulliyyah of Islamic Revealed Knowledge and Human Sciences (KIRKHS) International Islamic University Malaysia , Kuala Lumpur, Malaysia. *

**Keywords:** Urban women, health, cervical cancer, Pap test, Bangladesh

## Abstract

Each year, many countries from developed world publishes reports on early cancer detection; which is absolutely absent in most developing countries like Bangladesh.Very limited evidence is found on the role and acceptance of Pap test among the women of Bangladesh in determining cervical cancer. More research and updates are needed relating Pap test in early detection of cervical cancer. Thus the purpose of this study is set to assess the opinions of Bangladeshiurban womentowardsthe Pap test. A questionnaire-based survey of 400 Bangladeshi urban women was evaluated by on their socio-demographic characteristics, knowledgeand attitudes towards Pap testing. In general, the findings reveal that respondents havea good understanding of thepurpose of Pap test screening with 3.92 (Mean score). With 3.54 Mean score,the respondents believed that Pap tests are recommended to women who are married and with 3.45 mean score women believed that Pap tests arerecommended only to those who have children. Generally, respondents possess good knowledge of Pap test and its purpose. These findings can be used in identifying prospect cervical cancer screening significance populations and trend for future intrusion.

## Introduction

In the coming decades, cancer is foreseen to be a more significant cause of mortality in Bangladesh. In Bangladesh the projected frequency of new cancer cases will be 21.4 million by 2030 (Hussain and Sullivan, 2013). Cervical cancer ranks as the most prevailing cancer among Bangladeshi females (Ahmed and Rahman, 2008).

Despite advances in screening and treatment during the past several decades, cervical cancer remains a major health problem for Bangladeshi women. The reason is, many women have never undergone a Pap test procedure, or are not tested regularly. Like other less developed countries, low socio-economic status, poverty and lack of knowledge are considered as the reasons for the low test rates on Bangladeshi women (Austin et al., 2002). 

Recent publications claimed that Pap tests have been credibly tested to reduce mortality due to cervical cancer. Scientific research also claimed that it takes several years for cervical cancer cells to form in a human body. Regular screening through Pap test can prevent death from cervical cancer due to early detection(Upendram et al., 2017). For that,existing research advocated women be educated with regards to health education, counselling, outreach programs or community-based interventions (Wong et al., 2008). 

Existing literatures also claimed that early detection and screening services among women of less developed countries are profoundly influenced by their cultural beliefs and norms (Johnston et al., 2004; Wong et al., 2008). However, recent publications on health proposed to increase screening rates regardless of women’s socio-demographic differences (Mohamad Diah et al., 2015; Margolis et al., 1998; Ahmed and Rahman, 2008).

Women of Bangladesh come for diagnosis and treatment usually whenit is too late (Nessa et al., 2013). This cause the cervical cancer to be detected at a much later stage which may be too late for treatment. This is the reason why approximately 18,000 of Bangladeshi women reported new cases of cervical cancer on annual basis and out of which amounting to over 10,000 women die from it. “According to hospital records in Bangladesh, it constitutes about 22-29% of all female cancers”. “Two-third (69.2%) of the women referred for cervical cancer screening awareof cervical cancer and half of the women (47.4%) know about prevention of the disease” (Nessa et al., 2013). 

A recent study shows that the risk factors of cervical cancer include early age during first marriage, the increasing number of marriage, poor personal hygiene, multiple full-term pregnancies, early age at first intercourse, and multiple sexual partners of the women.Strategies should be implemented to amplify screening rates (Margolis et al., 1998). The objective of this current study is to know the opinions of Bangladeshi women in conducting Pap tests.

*Problem statement*

Cancer becomes a major public health concern throughout the world (Jemal et al., 2005). Cervical cancer is the second most common cancers among women and the most common cancer in the female of developing countries (Clifford et al., 2003). 

Every day, approximately 740 deaths occur from cervical cancer worldwide. The rate is not equally distributed in the world. Like many other discrepancies between developing and developed countries, mortality rate from cervical cancer is very obvious It is reported that 85% of death from this cancer happens in low and middle-income countries (LaVigne et al., 2017). 

Socio-economic status is the major factor for women health rationalisation. Women with high income and/orrich countries live longer and suffer less illness than poorwomen of less developed countries (World Health Organization, 2009). Though cervical cancer is preventable but like many other developing countries, Bangladeshi women also suffer and experience early death due to cervical cancer. Cervical cancer can be prevented by Pap screening.

In developing countries, out of a number of women diagnosed with cervical cancer, around 85% of women face death which is a big concern for social researchers and policymakers. Secondary sources (Nessa et al., 2013) claimed that around 22-29% of Bangladeshi women suffer from cervical cancer. Though Bangladesh offer screening program but investment in screening, diagnosis and treatment are rather inadequate in Bangladesh (Ferdausi et al., 2017). 

In Bangladesh, cervical cancer is comparatively a neglected disease in terms of promotion, screening and prevention. Public awareness is needed to create awareness with regards to cervical cancer. This situation put the researchers to put theeffort in identifying the level of knowledge and opinions of Bangladeshi women. This is why the objective is set to gain an understanding of Bangladeshi women’s opinions about cervical cancer, and to identify major concern on early screening for cervical cancer.

*Significance of the study*

Few researchers of Bangladesh have conducted their research on women health in general and Pap screening in specific (Begum and Hossain, 2006). Few others relate Pap screening from South Asian perspectives (Sankaranarayanan et al., 2008). Eventhough the death rate from cervical cancer increased in recent times but there is no significant academic research in Bangladesh focusing on women health and Pap screening. In this context, this research has broader appeals and this research will benefit different stakeholders of the society such as:

Firstly, this research identified the level of knowledge and awareness of Bangladeshi women with regards toPap test. This will assist policymakers to formulate required policies to disseminate more knowledge and increase awareness among Bangladeshi women. 

Secondly, the research conceptualised the socio-demographic factors of the respondents that have a significant impact on their knowledge and attitude about Pap test. This research thus will help the government to take initiatives in fulfilling the gap between different demographic groups. 

Thirdly, being the members of a developing country, women of Bangladesh suffer from various health diseases which requires much attention to be researched. This research will work as a guide for future academic research which is eventually a great contribution in the academic domain.

*Women Status in Bangladesh*

Bangladesh is one of the densely populated and developing countries of the world. Though Bangladesh achieved its success in declining its population howeverthe bitter truth is that around 2.0 million populations are being added to the mainstream population annually. This situation positions around 29% of the total Bangladeshi people to live earning less than US $ 1.00 per day (Ahmed, et al., 2005). Collin et al., (2007) stated that Bangladeshi women do not get proper coverage of health care service and this access varies depending on their socio-economic position in the society.

Bangladesh is a conservative country (Ahmed, 2015), predominantly having a Muslim majority population. Culturally the practice of purdah (full body covered up dress for Muslim women) is typical and considered a norm in this country. However, itis also true that purdah culture differs depending on their socio-economic position in the society (Islam et al., 2002). 

In South Asia, sharing advantages from household resources are biased systematically (Mukherjee et al., 2017). In most of the South Asian countries, including Bangladesh, higher education varies disproportionately among the population. The rich acquire significant education opportunities compared to the poor (Ilie and Rose, 2017). 

It is very much evident that Bangladeshi men have controlling attitudes and behaviour toward women. In getting health care support, for example, hospital admissions and diagnosis or treatment, women need socio-psychological and even economical support from the men. Moreover, culturally as the women tend to be conservative, many of them feel wary to go to hospital. This leads to discomfort if they are in need to visit any male doctor (Shahabuddin et al., 2017). 

*Literature Review*

Cervical cancer occurs during the reproductive age of women. To prevent this, the initial screening should be after the starting age of sexual intercourse (Begum and Hossain, 2006). A survey based study suggested that socio-culturally, Asian women are less into Pap screening compared to the non-Asian and White women (Marlow et al., 2015). This research finding supplements that Asian women are less interested in screening unless they suffer from related symptoms. A further study is needed to know the attitudes and opinions of Bangladeshi women towards pap screening. 

Socio-economic status of women isthe key indicator in determining women attitude towards pap screening (Moser et al., 2009). Literature shows that fatalistic attitudes, lack of family support, lack of information and awareness are the major issues associated with Pap screening among women (Behbakht et al., 2004). This research was conducted with a group of women diagnosed with cervical cancer and it shows that there is lack of information and awareness with regards to a Pap screening due to their low educational level and less health care support incites their attitude towards pap screening (Behbakht et al., 2004). While this study has explored the factors associated with pap screening and women with cervical cancer, research is needed to understand the factors associated with women from random selection. 

Even though America is a developed country but it has failed to provide equal opportunity to all its citizens (Tremblay, 2019). A number of research had shown that Hispanic, Asian or African female living in America are less concerned about Pap screening due to their low health care insurance, poverty and lack of education (Hawkes et al., 2002). Ansink et al., (2008) did qualitative research to study the level of awareness and attitudes of women towards Pap screening. The research found that the participants are informed about cervical cancer and its severity but their level of awareness is not significant even though they are found to be prone to social issues. Social barriers like limited health insurance and high expenseof treatment were found to be significant in screening. As this research facilitates to identify a number of factors in determining attitudes towards pap screening, a quantitative work is needed to assess the level of knowledge and attitudes towards pap screening. 

A similar result was found in another research conducted in Canada. The specific research revealed that women of mainland Nova Scotia and Cape Breton differed significantly in having screening by age, income, the origin of country, race, and location of their current residence. McFarland (2003) did a research with women in Botswana. Though cervical cancer is one of the most common cancers over there, the research revealed that their knowledge and awareness level of Pap screen is very low. This research used a Health model to explore the knowledge, awareness, and beliefs of cervical cancer and thus of Pap test screening. For this research, researchers used network sampling to get 30 women as their sample. The result disclosed that the knowledge and awareness of both cervical cancer and Pap test was found to be low among the low-income respondents. These researches were done in developed societies and now it is needed to assess the level of knowledge and attitudes towards pap screening in a less developed society like Bangladesh. 

Another qualitative study was conducted by Wong et al., (2008) with Malaysian women. The women were aged between 21 to 56 and they specifically chose women who had never gone through a Pap test. Awkwardness was found as one of the reasons of not having Pap testduringtheir life. This current study now aimed to study another Muslim majority country like Bangladesh to assess the opinion of women about Pap test. However, unlike Wong et al., (2008), this present research aimed both with screening and without screening women as the research sample by using quantitative data.

Though the research of Marlow et al.,(2015) found that Asian women are less concerned in pap screening unless they are affected by some symptoms, the similar result is found in works of Nielson et al., (2004) with non-Asian respondents. By using ethnography methodology, these researchers found that as long as there is no symptom, the participants did not go for health screening. As the reason for their unwillingness, these respondents mostly cited their busy schedule (Nielsen et al., 2004) 

Research findings (Jacobsen and Jacobsen, 2011) show that campaignscan increase the rate of immediate screening. The role of media in increasing health awareness level is recommended by many researchers. Grilli et al.,(2002) talked about the role of mass media in enhancing health-related awareness among the people. They wrote, “mass media information on health-related issues may induce changes in health services, utilisation, both through planned campaigns and unplanned coverage” (2002).

From the above comprehensive literature review, it is clear that cervical cancer is one of the major concerns for women health. The risk of cervical cancer can be reduced with regular screening. However, there is still a big vacuum in empirical research in women health issues in Bangladesh, particularly by relating cervical cancer and Pap test. This research attempts to fulfil this gap. 

*Conceptual Framework*

Awareness increases people’s skills in understanding various aspects of social, political and economic issues (Mahmud et al, 2014). In examining the issue of Pap test; the influence of socio-economic factors needs to be understood. More specifically, education level is useful in having Pap test. A number of previous researches (Ojanuga and Gilbert, 1992) disclosed that educated women are more concerned about their own health compared to the illiterate and less educated women. So to increase the health education among the female in developing countries like Bangladesh both formal (such as school) and informal (such as media campaign) education is required. Likewise, secondary sources claimed that marital status has significant impact inobtaining health care services. In his work, Umberson (1992), showed that mortality rates among the married women are less than the unmarried womensince they are moreattentivewith regards to their health conditions. Similarly, secondary sources (Mandelblatt et al, 1999; Kabir et al, 2018) found a relationship between age and receiving health care services among women. They showed that elderly women who are more than 65 and above in age, are less likely to undergohealth care screeningthan younger women. Influence of having children and getting health care services among twomen cannot be ignored. It is important to note that due to the lack of information about Pap testand its benefits, women of Bangladesh do not get their screening on time. The campaign from government, hospitals and the media is very limited which hinders the women to know the latest advancement of treatment. Moreover, once they become sick and visit the doctors, then, onlythe women are informed about Pap test. 

## Materials and Methods

This section focused on the methods used in this study. Methods section is divided into three sub-sections. Section one shed lights mainly on location and time of the study, target groups of the study, sampling and data collection procedure of the study. Section two highlighted the descriptive analysis used for describing the socio-economic characteristics of the respondents. Finally, section three emphasised on the opinions of the respondents regarding the Pap test. 

*Section one*

*a. Location and time of the study*

This study was conducted in three (3) hospitals of Dhaka city named,Bangabandhu Sheikh Mujib Medical University (BSMMU) hospital, Dhaka Medical College Hospital and Shaheed Suhrawardy Medical College. The data collection for this study started in July 2016 and continued till December 2016. 

*b. Target groups of the study*

The target group of this study is women who live in the urban areas of Dhaka city within the range of 20-79 years of age.

*c. Sampling*

Bangabandhu Sheikh Mujib Medical University (BSMMU) hospital, Dhaka Medical College Hospital and Shaheed Suhrawardy Medical Collegewere chosen purposively, because: 

i. Pap test is available in these hospitals. 

ii. Moreover, these three hospitals are government operated hospitals where patients of different socio-economic background are provided with health care services. 

A list of women who registered for medical services from these three hospitals during July to December 2016 was collected from the administrative offices of the above mentioned hospitals. Out of 1398 women, a total of 400 women were selected as sample using simple random sampling technique at the 95% confidence interval conceiving 4% of error. 

*d. Data collection*

Primary data was collected from the selected samples using questionnaire(the questionnaire was developed by using many components from Mohamad Diahet al., (2015). Data were mainly collected on the following aspects:

a. Socio-economic profile of the respondents

b. The practice related to Pap test

c. Opinions of the respondents on Pap test 

d. Analytical techniques

Quantitative methods were used to analyse the collected data. Descriptive (mean, percentage, standard deviation) analysis was used to describe the socio-economic characteristics of the respondents. 5-points Likert scales were used to assess the opinions of the respondents regarding Pap test. Moreover, Chi- square test was conducted to examine the relationship between socio-economic factors and attitude of practising Pap test. 

## Results

Based on descriptive statistics analyses conducted for Bangladeshi women, the following demographic information was found to be associated with Pap screening. Number of demographic issues like age, marital status, employment status, highest level of education, having/ had children, the number of children, having/had pap test,the number of Pap test, the place ofthe test was conducted,the result of the test, conducted by whom, theplace of knowledge of Pap test are stated.

**Figure 1:**
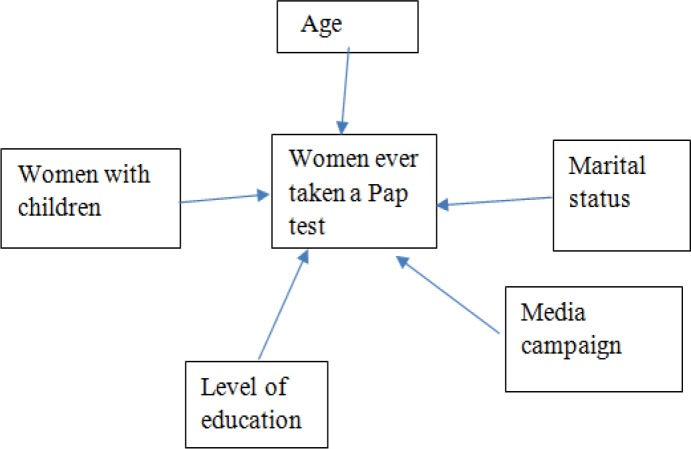
Conceptual Framework for Opinions of the Urban Women on Pap Test

**Table 1:** Profile of the Respondents

Item	Frequency	Percentage (%)
Age		
20-29	150	37.5
30-39	136	34.00
40-49	75	18.75
50-59	33	8.30
60-69	5	1.30
Prefer not to disclose	1	0.30
Marital status		
Single	58	14.50
Married	314	78.50
Divorced	4	1.00
Widowed	22	5.50
Prefer not to disclose	2	0.50
Employment status		
Full time	84	21.00
Part time	9	2.25
Self-employed	3	0.75
Retired	64	16.00
Unemployed	237	59.25
Prefer not to disclose	3	0.75
Highest level of education		
Primary education	155	38.75
Secondary education	131	32.75
Diploma	9	2.25
Undergraduate	54	13.50
Postgraduate	49	12.25
Prefer not to disclose	2	0.50
Any children		
Yes	317	79.25
No	82	20.50
Prefer not to disclose	1	0.25
If yes, number of children		
1-2	206	64.32
3-4	103	32.49
5 and above years)	8	2.52

Source, Survey, 2016

**Table 2:** Practice Related to Pap Test

Items	Frequency	Percentage (%)
Have you ever taken a Pap test?		
Yes	167	41.8
No	232	58.00
Prefer not to disclose	1	0.30
If yes, how many times		
1-2	125	74.85
3-4	25	14.97
5 and above	8	4.79
Prefer not to disclose	9	5.38
Result of the last Pap test		
Positive	119	71.26
Negative	41	24.55
Prefer not to disclose	7	4.19
Place of test conduct		
Private hospital	21	12.57
Government hospital	133	79.64
Private clinic	2	1.19
Government clinic	5	2.99
Prefer not to disclose	6	3.59
Pap test conducted by		
Male doctor	1	0.59
Female doctor	158	94.61
Others	8	4.79
Source of knowledge about Pap test		
Magazines	1	0.62
TV	0	0
Radio	1	0.62
Clinics	5	3.11
Hospital	141	87.58
Friends	1	0.62
Family	9	5.60
Prefer not to disclose	3	1.86

Source, Survey, 2016

**Table 3:** Result of Cross-Tabulation with “Have You Ever Taken a Pap Test?”

Items	Have you ever taken a Pap test?			
Marital status	Yes	No	Total	Sig
Single	0	58	58	Pearson Chi-Square: 0.000
Married	151	163	314	
Divorced	3	1	4	
Widowed	13	9	22	
Age (in years)				
20-29	38	112	150	Pearson Chi-Square: 0.000
30-39	60	76	136	
40-49	43	32	75	
50-59	22	11	33	
60-69	4	1	5	
Employment status				
Full time	32	52	84	Pearson Chi-Square: 0.541
Part time	3	6	9	
Self-employed	1	2	3	
Retired	23	41	64	
Unemployed	108	129	237	
Highest level of education				
Primary education	66	89	155	Pearson Chi-Square: 0.000
Secondary education	73	58	131	
Diploma	5	4	9	
Undergraduate	3	51	54	
Postgraduate	20	29	49	
Any children				
Yes	161	156	317	Sig.lev: Pearson Chi-square: 0.000
No	6	76	82	

Source, Survey, 2016

**Table 4:** Opinions of the Respondents on Pap Test

Statements	Mean	Std. Deviation
S1. A Pap test reduces your risk for cervical cancer.	3.82	0.64
S2. A Pap test can detect the early development of cervical cancer.	3.92	2.11
S3. Women ages 21 to 65 should get Pap test as part of their routine health care.	3.61	0.65
S4. You must have a Pap test every 3 years.	3.7	1.61
S5. I am aware of the risks of cervical cancer.	3.8	0.61
S6. Pap testsare recommended to women who are married.	3.54	0.85
S7. Pap tests are recommended only to those who have had children.	3.45	0.91
S8. If you are unmarried, you do not need a Pap test.	2.45	1.14
S9. Women who have gone through menopause do not need a Pap test.	2.81	1.02
S10. Poor cleanliness is a risk factor for cervical cancer.	3.82	1.62
S11. An abnormal Pap test is positive for cervical cancer.	3.75	2.03
S12. A healthy life means a Pap test is not necessary.	2.11	0.95
S13. Only women who have a family history of cervical cancer should get a Pap test.	2.16	0.84
S14. A Pap test is a preventive measure for cervical cancer.	3.77	0.678

Source, Survey, 2016

**Table 5:** Attitudes Towards Pap Test

Statements	Mean	Std. Deviation
S1. A Pap test should only be conducted by a female doctor.	3.81	0.816
S2. Getting a Pap test is painful.	3.35	0.878
S3. Getting a Pap test is embarrassing.	2.58	0.994
S4. I do not mind a Pap test being conducted by a male doctor.	2.29	0.979
S5. Getting a Pap test is costly.	2.24	1.06
S6. My family encourages me to have a Pap test.	3.21	0.958
S7. Neither my mother and nor I had a pap test.	2.73	1.06
S8. A Pap test is a private matter.	2.42	1.02
S9. Advertisement for a free Pap test would motivate me to get one.	3.76	2.69
S10. A Pap test will cause an unmarried woman to lose her virginity.	2.72	0.976
S11. I am not comfortable with any medical procedures concerning my vagina.	2.65	1.06

Source, Survey, 2016

*Profile of the respondents*

Table 1 describes the demographic information of the respondents. Here the data shows that 90.25% of the respondents are within the age of 20 to 49. The women participated as the respondents of this research aremostly (78.5%) married. Moreover, more than half (59.25%) of the respondents are found as unemployed. These respondents are found very less educated as most (71.5%) of the respondents’ had only secondary or less than secondary education. Out of all these respondents, 79.25% of respondents have children of their own. Most (64.32%) of the respondents have 1-2 children. 

*Practice related to Pap test*

In Table 2, the information with regards to Pap test practice is shown. At the beginning of the table, it was shown whether they had ever taken Pap test or not. The data shows that 58% of the respondents never took a Pap test; however 41.8% of the respondents told that they had experience with the Pap test. 74.85% of the respondents went through Pap test with a maximum of 2 times in their life so far. The respondents who had Pap test, 71.26% of them got a positive result in it which means they are currently suffering from cervical related diseases. It is found that most (79.64%) of the respondents had taken their test in Government hospitals and 94.61% of the respondents with Pap test conducted their test with female doctors. The interesting findings of this section are the other sources except for Hospitals in disseminating knowledge about Pap test are very negligible. The findings show 87.58% of the respondents knew about Pap test from their hospitals after being infected with various cervical related complications. 

A cross tabulation was done where “have you ever taken a Pap test” was found significantly correlated with marital status. The significant level of Pearson Chi-square was .000. It shows that unmarried women had never taken Pap test, whereas, the married women are likely to have a Pap test. Like marital status, some other variables like age and education was also found significantly correlated with “have you ever taken a Pap test”. The significant level of all these three variables Pearson Chi-square was .000. However, this research did not get any significant relation of employment status with “have you ever taken a Pap test”. The significant level of Pearson Chi-square was found for employment status was .541.

*Opinions of the respondents on Pap test*

Fourteen statementshighlighted to draw participants’ opinions and understanding concerning Pap test. The study aims to find out Bangladeshi women’s opinions regarding Pap test as a screening process. 

Respondents’ opinion about Pap test was measured by Mean score and Standard Deviation. The mean score of “A Pap test can detect the early development of cervical cancer” was 3.92 which mean they are very close to agreeing to the fact. Like that some other facts are also very close to agreeing; such as mean score 3.82 of “A Pap test reduces your risk for cervical cancer”. “I am aware of the risks of cervical cancer” was with a mean score of 3.80 which reflects that respondents are about to agree with it. Similarly, mean score (3.77) of “a Pap test is a preventive measure for cervical cancer” is also very close to 4 in its scale. Though the mean score of a number of statements is very close to the point “agree” but none of the scores crossed the scale. However few statements are found with “disagree” mean score. A statement was “If you are unmarried, you do not need a Pap test”, which got a mean score of 2.45 which is a clear reflection of their disagreement. Like another statement, “Women who have gone through menopause do not need a Pap test”, had got a mean score with disagreement (2.81) value. With a mean score of 2.11, respondents were about to strongly disagree on “A healthy life means a Pap test is not necessary”. Like some other statements, “only women who have a family history of cervical cancer should get a Pap test” also receives a low mean score of 2.16 which is their clear disagreement.

## Discussion

Respondents’ attitude towards Pap test was measured by Mean score and Standard Deviation. The mean score of the statement “Getting a Pap test is embarrassing” is 2.58 which reflect their disagreement with the statement. Likewise, the respondents’ showed their disagreement with the statement “I do not mind a Pap test being conducted by a male doctor” by giving a mean score of 2.29 which tell that for treatment purpose they do not mind to go even to a male doctor. Similarly, the respondents do not consider of having Pap test as very costly. As the Mean score for “Getting a Pap test is costly” and is 2.24 as many government hospital provides this service at no cost. Though many of the respondents were less educated however the mean of “Neither my mother and nor I had a pap test” was 2.73 which shows their clear disagreement with the statement. Like the previous section of opinions, in this section also, this study did not get any score of 4 and above.

Bangladesh is a developing country which almost half of its population is female. To make a country self-dependent and economically prosperous, it is very much needed to focus onthe female population as well. Among many other common diseases, cervical cancer is considered one of the common diseases for women. Women canavoid the disease if they take the screening test before cancerreach its severe stage. In Bangladesh, government hospitals in each district are giving VIA (visual inspections with acetic acid)test which primarily can detect the abnormality of a vagina. However, Pap screening is even more recognised worldwide for its better screening capacity. 

It is found that most of the women who had come to the hospitals for Pap screening are informed about Pap test by their physician. Other mediums are very weak in disseminating awareness about cervical cancer and its precautions screening. This screening test is practised mostly by the married women with children. 

Bangladesh society typically is considered as a conservative society. Any treatment which deals with the female vagina is consideredsensitive for any female from any social background. However, education and women empowerment in societal level cause a significant change in people perception in general, women perception in specific. This is found true in this study too. Though these female is from a conservative Bengali society, they do not think that taking a test in vagina for health checking purpose is embarrassing at all.

*Limitations of the study*

Although this research has significant contributions in the academic and practical sector, this is also true that the research has a number of limitations. Such as, only quantitative method has been used in this research. It could be a comprehensive research work if a qualitative method can be used together with a quantitative method. Moreover, if the opinions of males can be taken into account, then it could be holistic research. In addition to these, research focus only on urban women can be taken as a research limitation of this study. 

*Conclusion and implications*

Cervical cancer is one of the neglected diseases in Bangladesh. Promotions, campaigns, screenings are overlooked in this country due to the lack of awarenessabout this disease. Public awareness is much needed to reduce the number of deaths from cervical cancer.Both government and non-government organizations should work together to make Bangladeshi females more aware and knowledgeable about Pap screening. 

If their health is not treated properly, Bangladesh will suffer economically, socially and culturally. Cervical cancer among women can be a threat for the development of Bangladesh society. Besides physicians, other social media like television, radio and other social networks should work together in disseminating knowledge about Pap test. To increase the pap screening awareness, femalesshould be more educated as education provides more knowledge, awareness, and information for female health issues. This research is academic research to know opinions of Bangladeshi women about Pap test. This research, thus, will advocate positive in policies’development in taking Pap test. 
